# Application of the augmented competing stimulus assessment to identify and establish competing self‐restraint items

**DOI:** 10.1002/jaba.70040

**Published:** 2025-11-03

**Authors:** Michelle A. Frank‐Crawford, Louis P. Hagopian, Jonathan D. Schmidt, Griffin W. Rooker, Drew E. Piersma, Ryan Benson

**Affiliations:** ^1^ Department of Behavioral Psychology Kennedy Krieger Institute Baltimore MD USA; ^2^ Department of Psychiatry and Behavioral Science Johns Hopkins University, School of Medicine Baltimore MD USA

**Keywords:** automatically maintained self‐injurious behavior, delayed hyperalgesia, self‐restraint, Subtype 3

## Abstract

Automatically maintained self‐injurious behavior (SIB) sometimes co‐occurs with self‐restraint, a self‐limiting behavior that impedes SIB and can be maladaptive (e.g., hinders functional skills and movement). The presence of self‐restraint suggests SIB produces aversive consequences, which self‐restraint limits. We conducted a prospective consecutive controlled case series study of five individuals with Subtype 3 automatically maintained SIB where we applied the augmented competing stimulus assessment to identify and establish alternative self‐restraint items to compete with existing forms of self‐restraint. At least one high‐competition item that produced an 80% or greater reduction in self‐restraint and SIB without disrupting toy engagement was identified for the four participants who completed assessment. We discuss the need for additional research on this procedure and how competing self‐restraint items can be used in combination with competing stimuli and tasks to address SIB and self‐restraint. We also discuss some avenues for research that is directed at understanding the mechanisms of self‐restraint.

Self‐injurious behavior (SIB) is sometimes associated with self‐restraint, which can generally be defined as behavior that may be incompatible with or physically impede SIB. Although self‐restraint is seemingly protective and preferable over SIB, it can interfere with adaptive behavior or produce injury (Foxx & Dufrense, 1984; Smith et al., 1992; see also Furniss & Biswas, 2020). Estimates on the overall prevalence of self‐restraint among individuals with intellectual and developmental disabilities vary from 4% to 76.14% (e.g., Marlow et al., 2024; Oliver et al., 2003; for a summary, see Furniss & Biswas, 2020). At least four topographical categories of self‐restraint have been described: (a) entwining extremities using clothing or other objects; (b) using one's own body to restrict moving by clasping hands, wedging hands under one's legs, or assuming positions that restrict movement; (c) requesting materials to use to restrain oneself, seeking mechanical restraint, or holding items; and (d) clinging to others, hand holding, or requesting to be held in a way that restricts movement (i.e., “recruited restraint;” Isley et al., 1991; Marlow et al., 2024; Oliver et al., 2003). Recently, Marlow et al. (2024) found that the most commonly reported topographies of self‐restraint include holding or squeezing objects and recruited restraint, both reported to occur among 32% of the population sampled.

Self‐restraint appears to be more common among individuals with intellectual and developmental disabilities who engage in automatically maintained SIB, but it can also occur among individuals with socially maintained SIB (Derby et al., 1996; Smith et al., 1996). In a consecutive controlled case series study of 152 cases with SIB, Iwata et al. (1994) reported that 7.24% (*n* = 11) of their total sample engaged in self‐restraint. However, it is not known what proportion of those with self‐restraint engaged in automatically maintained SIB. In a more recent consecutive controlled case series study of 39 individuals with automatically maintained SIB, 20.51% (*n* = 8) engaged in self‐restraint in over 25% of intervals of the no‐interaction condition of the functional analysis (Hagopian et al., 2015).

Evidence of the functional relation between self‐restraint and SIB comes from case reports examining these behaviors across different conditions (e.g., Derby et al., 1996; Fisher & Iwata, 1996; Jennett, Hagopian, & Beaulieu, 2011; Kerth et al., 2009; Lerman et al., 1994; Pace et al., 1986; Rooker & Roscoe, 2005; Scheithauer et al., 2015; Smith et al., 1996). Self‐restraint and SIB often vary inversely with one another, although they are not necessarily incompatible. When self‐restraint is allowed, SIB is often reduced, and conversely, SIB sometimes increases when self‐restraint is limited (e.g., Fisher & Iwata, 1996; Hagopian et al., 2015; Scheithauer et al., 2015; Silverman et al., 1984). Some individuals also reportedly seek the application of mechanical restraint devices, and the signaled removal of such devices can result in indicators of emotional distress (e.g., Jennett, Hagopian, & Beaulieu, 2011; see Romanczyk et al., 1992, for a discussion). Based on these and similar findings, it has been hypothesized that the presence of self‐restraint suggests that SIB produces aversive consequences, which self‐restraint may have historically avoided or minimized (see Fisher & Iwata, 1996). Thus, self‐restraint may be evoked or occasioned by the aversive consequences of SIB and negatively reinforced by avoidance of those aversive consequences.^1^

There is evidence for subtypes of automatically maintained SIB based on response features thought to be linked to distinct behavioral mechanisms (Hagopian et al., 2015, 2017). Self‐restraint is a defining feature for one such subtype. Specifically, Subtype 3 is characterized by the presence of self‐restraint based on the hypothesized mechanism described above: that its occurrence has been historically negatively reinforced by the prevention or minimization of SIB. Research has also suggested that SIB classified as Subtype 3 is highly resistant to treatment using reinforcement alone, often necessitating mechanical restraint and protective equipment (Hagopian et al., 2015).

Unfortunately, the literature on the treatment of self‐restraint is quite limited and somewhat dated (for reviews, see Furniss & Biswas, 2020; Marlow et al., 2024). In fact, the most recent study published in the *Journal of Applied Behavior Analysis* on the treatment of self‐restraint appeared 10 years ago (Scheithauer et al., 2015). A variety of treatment approaches have been examined, such as using protective equipment or mechanical restraint (e.g., Oliver et al., 1998; Powers et al., 2007), fading the size of items used to engage in self‐restraint (e.g., Banda et al., 2012; Foxx & Dufrense, 1984; Lerman et al., 1994; Pace et al., 1986), and providing reinforcement for the absence of SIB (e.g., Banda et al., 2012; Foxx & Dufrense, 1984). Another approach to the treatment of self‐restraint involves introducing alternative items with which the individual can self‐restrain in a manner viewed as more socially acceptable or that may interfere less with functional activities (e.g., Dawson et al., 2025; Rooker & Roscoe, 2005; Scheithauer et al., 2015; Silverman et al., 1984; Vollmer & Vorndran, 1998).

Rooker and Roscoe (2005) were the first to describe a systematic approach to selecting such alternative self‐restraint items. The authors reported on an individual whose self‐restraint involved positioning items, such as a life vest or stuffed animals, between his chin and shoulder. After conducting a preference assessment of alternative items that could be similarly positioned, the experimenters conducted a competing stimulus assessment (CSA) to examine how access to each item affected SIB. The participant voluntarily engaged with every item, and two airplane neck pillows were associated with no SIB. This study represents a noteworthy technological advancement, as it was the first to employ CSA methodology to empirically select alternative, competing self‐restraint items based on the extent to which they reduced SIB. Unlike the typical CSA, the items assessed were not leisure items hypothesized to compete with reinforcers maintaining SIB. Rather, they were items that could replace the life vest and stuffed animals. The authors viewed these alternative items as more socially acceptable than the existing items used to engage in self‐restraint.

More recently, Hagopian et al. (2020) described outcomes from an augmented CSA (A‐CSA) for six individuals with automatically maintained SIB, two of whom engaged in Subtype 3 SIB (P1 and P4). During the initial free‐access condition, the researchers identified only one high‐competition (HC) stimulus (i.e., that produced an 80% reduction in self‐restraint and SIB) for P1 and no HC stimuli for P4. Prompted engagement and response blocking with redirection tactics were then systematically employed to increase engagement with test stimuli and to decrease SIB and self‐restraint. When these tactics were withdrawn in the repeated free‐access condition, the number of identified HC stimuli increased to seven for P1 and four for P4. Frank‐Crawford et al. (2023) replicated these findings with four additional participants with SIB classified as Subtype 3.

The test stimuli evaluated in the A‐CSA by Hagopian et al. (2020) and Frank‐Crawford et al. (2023) included various leisure items. Generally, reductions in challenging behavior during a CSA are thought to result from reinforcer competition (or substitution; see Haddock & Hagopian, 2020). For cases with self‐restraint, it is possible that HC stimuli decreased SIB through reinforcer competition, which in turn decreased the establishing operation for self‐restraint. An alternative, and perhaps complimentary, approach would be to identify items that directly compete with or replace self‐restraint to limit SIB (and the putative aversive consequences it produces). This is similar to the approach taken by Rooker and Roscoe (2005) in that they conducted a CSA of alternative self‐restraint items. However, Rooker and Roscoe only assessed the effects of the items on SIB; they did not measure self‐restraint. Moreover, these studies did not assess the influence of the HC items on adaptive behavior, which is a critical consideration when addressing self‐restraint. Furthermore, there is a dearth of research on Subtype 3. To that end, the current study used the A‐CSA to identify and establish competing self‐restraint items associated with reductions in the existing form of self‐restraint and SIB, without interfering with an adaptive response (toy engagement), for five individuals with Subtype 3 automatically maintained SIB.

## METHOD

### Participants and setting

All participants had been admitted to an inpatient unit for the treatment of severe challenging behavior, and they were subsequently enrolled in a prospective research study investigating behavioral interventions for treatment‐resistant subtypes of automatically maintained SIB (Hagopian, 2019; Identification No. NCT03995966). Individuals with developmental disabilities between the ages of 4 and 25 years with automatically maintained SIB classified as Subtype 2 or 3 were eligible to participate in the study. A functional analysis (Iwata et al., 1982/1994) was conducted with each participant before enrollment in the study as part of routine clinical care on the inpatient unit; the functional analysis was completed under the direction of a doctoral‐level Board Certified Behavior Analyst. Five individuals who met criteria were recruited into the current study. The study received approval from an institutional review board and the participants' caregivers provided consent for participation. Functional analysis outcomes for these five participants indicated SIB was maintained by automatic reinforcement and classified as Subtype 3 according to the subtyping model described by Hagopian et al. (2015, 2017). Table 1 includes participant diagnoses, the Standard Scores from the Vineland Adaptive Behavior Scale, 3rd edition (Vineland‐3; Sparrow et al., 2016), and results of the Aberrant Behavior Checklist (Aman & Singh, 1986). Table 2 depicts the functional analysis outcomes, including the level of differentiation between the no‐interaction and toy play conditions and percentage of session with self‐restraint.

**TABLE 1:** Participant demographic information.

P	Diagnoses	Level of ID	Vineland‐3 Standard Scores				Aberrant Behavior Checklist Result				
			ABC	Comm	DLS	Soc	Irr	Leth	Stereo	Hyper	Inapp speech
Nash	ASD	Moderate	53	42	48	54	Clin Sig	Clin Sig	Clin Sig	Normal	Elevated
Jonah	ASD, SMD with SIB	Severe	21	20	23	20	Clin Sig	Elevated	Elevated	Clin Sig	Normal
Evan	ASD	Severe	33	20	44	24	Clin Sig	Clin Sig	Clin Sig	Normal	Clin Sig
Zuri	ASD, SMD with SIB, DICCD, Sotos Syndrome	Severe	‐	‐	‐	‐	Clin Sig	Clin Sig	Clin Sig	Elevated	Clin Sig
Daniel	ASD, SMD with SIB, DICCD, ADHD, DMDD	Moderate	41	21	51	36	Clin Sig	Clin Sig	Clin Sig	Elevated	Normal

*Note*: P = participant; ID = intellectual disability; ABC = Adaptive Behavior Composite; Comm = Communication Domain; DLS = Daily Living Skills Domain; Soc = Socialization Domain; Irr = Irritability subscale; Leth = Lethargy subscale; Stereo = Stereotypy subscale; Hyper = Hyperactivity subscale; Inapp Speech = Inappropriate Speech subscale; Clin Sig = clinically significant; ASD = autism spectrum disorder; SMD with SIB = stereotypic movement disorder with self‐injurious behavior; DICCD = disruptive, impulse control, and conduct disorders; ADHD = attention‐deficit/hyperactivity disorder; DMDD = disruptive mood dysregulation disorder.

**TABLE 2:** Functional analysis data.

P	Self‐injurious behavior			Self‐restraint	
	Toy Play	Alone/No Interaction	LOD	Toy Play (Control, no MR^a^)	Alone/No Interaction (MR^a^)
Nash	1.32	1.08	−22.22%	21.05%	50.85%
Jonah	1	1.37	27.01%	38.80%	70.44%
Evan	1.68	4.65	63.80%	21.17%	32.87%
Zuri	0	9.02	100%	97.50%	97.90%
Daniel	2.90	33.63	91.38%	3.50%	41.76%

*Note*: Values represent responses per minute unless denoted with % for percentage of session.

P = participant; LOD = level of differentiation; MR = mechanical restraint.

^a^ For Daniel, a separate analysis was conducted to determine Subtype 3; therefore, the data for restraint represent the percentage of session in which Daniel engaged in recruited restraint when mechanical restraint (MR) was not (Control) and was present.

Nash was a 10‐year‐old White non‐Hispanic male who communicated vocally using 1‐ to 2‐word phrases. His school reportedly applied a helmet and safety holds noncontingently to reduce his risk of injury from SIB, and his caregivers reported using arm wraps to protect his arms from self‐biting. He had a history of open wounds to his neck as well as lacerations, contusions, and concussions as a result of his SIB. During his admission functional analysis, he engaged in self‐restraint in 20%–90% of sessions.

Jonah was a 19‐year‐old male (race and ethnicity were not reported) who communicated using gestures and some signs. Prior to his admission, multiple safety procedures were reportedly tried, including posey mitts, helmets, and mechanical restraint, and safety holds. He had a history of bruising, edema, and lacerations to the head from SIB as well as hair loss. During his admission functional analysis, he engaged in self‐restraint in 45%–99% of sessions.

Evan was an 8‐year‐old White non‐Hispanic male who communicated using one‐word phrases and via an augmentative and alternative communication device. Prior to his admission, he wore a padded helmet across all waking hours with caregivers and intermittently at school. Caregivers reported that he was “attached” to his helmet and rarely took it off at home. School staff also reported that Evan engaged in increased levels of SIB if he could not touch or hold their hands. Evan had a history of bruising, edema, and lacerations to the head from head‐directed SIB and head banging. During his admission functional analysis, he engaged in self‐restraint in 0%–89% of sessions.

Zuri was a 10‐year‐old White non‐Hispanic female who communicated using gestures and some signs. Prior to her admission, she was reported to wear mechanical restraint in the form of rigid arm splints, several forms of protective equipment (padded gloves, a helmet with face shield, and foot guards), and padded pants. Zuri was legally blind in both eyes due to retinal detachment because of her head‐directed SIB. She had a history of contusions and lacerations, open wounds on hands and extremities from self‐biting, and swollen lips. Caregivers reported having to always be by her side and having to position their hands across her body. During her admission functional analysis, she engaged in self‐restraint in 0%–94% of sessions.

Daniel was a 9‐year‐old male (race and ethnicity were not reported) who communicated using gestures and some signs. Prior to his admission, he periodically wore a padded helmet to reduce his risk of injury to his head; he was reported to engage in high rates of SIB and recruited restraint in the form of hand holding by school staff. He had a history of bruising and cuts to his face, ears, chin, and knuckles from his SIB. During his admission functional analysis, he engaged in self‐restraint in 29%–60% of sessions.

Sessions were conducted in padded rooms (3 × 3 m) for Nash, Jonah, and Evan and a bedroom (3.50 × 3.50 m) for Zuri. For Daniel, the presession exposure to test items was conducted in an activity area (3.20 × 3.20 m). These five cases represent all the consecutively encountered cases who presented with Subtype 3 automatically maintained SIB at the time of this study, meaning that no cases that met the study inclusion criteria were excluded. Additional design, data‐analytic, and reporting elements characteristic of a prospective consecutive controlled case series were employed (see Hagopian, 2020).

### Response definitions and data collection

Nash's SIB included *skin‐directed SIB* (pinching, squeezing, picking, or scratching any part of his body, face, or throat with the exception of his neck), *head banging* (hitting his head against surfaces from a distance of 15.24 cm or greater), and *neck‐directed SIB* (pinching, squeezing, picking, or scratching any part of his neck). Nash's *self‐restraint* included laying or sitting on his arms or hands, lacing his fingers together, holding objects or his hands under objects, entwining his hands in his clothing, and holding onto others. Jonah's *head‐directed SIB* included hitting any part of his head, neck, or face with an open hand or closed fist and pulling his own hair. Jonah's *self‐restraint* included laying or sitting on his arms or hands, pushing his arms or hands against a surface, lacing his fingers together, entwining his hands in his clothing, placing his hands through the handles of a portable mat, and holding onto the therapist's hands or positioning the therapist's arm or body over his own arms. Evan's *head‐directed SIB* included hitting any part of the head or face with an open or closed fist, wrist, arm, or object; forcefully pressing his chin onto objects; and self‐hair pulling. Evan's *self‐restraint* included holding onto therapist's hands or positioning the therapist's arm or body over his own hands or arms. Zuri's *head‐directed SIB* included slapping or hitting any part of her head or face, chin pressing, eye poking, and hitting any part of her head or face with her knee or the area above or below the knee. Zuri's *body‐directed SIB* included hitting or kicking any part of her body, other than her face, with an open or closed hand and body slamming. Zuri also engaged in *skin‐directed SIB*, which included biting, scratching, pinching, or picking any part of her body. Finally, Zuri's *head banging* included hitting her head against surfaces from a distance of 15.24 cm or greater. Zuri's *self‐restraint* included laying or sitting on her arms or hands, tucking her arms behind her back, crossing her arms in her lap, lacing her fingers together, holding objects between her head and shoulder or head and chin, entwining her hands in her clothing, and holding onto therapist's hands or positioning the therapist's arm or body over her own arms. Daniel's *head‐directed SIB* included hitting any part of his head or face (aside from his chin) with an open or closed hand or object. Daniel's *body‐directed SIB* included hitting any part of the body with an open or closed fist or an object, body slamming, and scratching or picking at any part of his body. Finally, Daniel's *chin‐directed SIB* included hitting any part of his chin with one or two hands. Daniel's *self*‐*restraint* included laying or sitting on his arms or hands, positioning his arms under objects, crossing his arms in his lap, entwining his hands in his clothing, and holding onto therapist's hands or placing the therapist's arms over his shoulders.

*Test‐item use* was generally defined as use of the test item in the intended manner. Supporting Information A includes individualized definitions for test‐item use for each item. For example, use of the airplane pillow included the participant keeping the pillow around some portion of their neck. *Toy engagement* was defined as any attempt to independently manipulate the toys in a manner that was not destructive or harmful to the participant or others and excluded mere contact with the toy and physically prompted engagement during conditions with response promotion. An *HC item* was operationally defined as a test item (a) associated with an 80% or greater reduction in the percentage of 10‐s intervals with SIB and self‐restraint relative to the control trial and (b) for which toy engagement was not disrupted by more than 10% relative to the control trial or remained above 80% of the session.

Trained observers used laptop computers to record either frequency or duration data for all target behaviors using the program BDataPro (Bullock et al., 2017). Across all participants, frequency data were recorded for all topographies of SIB; for Nash, Jonah, and Evan, frequency data were also recorded for blocked attempts to engage in SIB, self‐restraint, or recruited restraint (attempts were not recorded for Zuri as she did not experience the response blocking condition). Additionally, duration data were collected for self‐restraint and recruited restraint as well as for test‐item use and toy engagement. The participants often alternated between engaging in SIB and self‐restraint within trials. In addition, self‐restraint and SIB were not necessarily mutually exclusive; thus, participants sometimes engaged in these responses simultaneously (e.g., a participant could sit on their hands while also kicking their own shin). Therefore, we used an aggregate measure of self‐restraint and SIB by calculating the percentage of 10‐s intervals in which either response occurred (or was blocked) by dividing the number of 10‐s intervals during which either occurred (or was blocked) by the total number of intervals in the trial. The ratio was multiplied by 100 to calculate the percentage of 10‐s intervals in which either self‐restraint or SIB occurred. Supporting Information B, C, D, and E include the A‐CSA outcomes for SIB and self‐restraint (and blocking when applicable) graphed separately. Duration data on toy engagement was converted to the percentage of session by dividing the total number of seconds of toy engagement by the total duration of session in seconds and multiplying the ratio by 100.

### Interobserver agreement and procedural fidelity

A secondary observer independently and simultaneously collected data during a mean of 57.06% (range: 35.71%–67.95%) of sessions across participants. For all sessions, total session time was divided into consecutive 10‐s intervals and data were compared on an interval‐by‐interval basis. Partial interval agreement (Mudford et al., 2009) was calculated for each measure by dividing the lower number of responses (either frequency or duration) by the higher number of responses (either frequency or duration) for each interval. These values were then summed across all 10‐s intervals and divided by the total number of intervals. The quotient was then converted to a percentage by multiplying the value by 100. Mean agreement coefficients across participants were 98.39% (range: 50%–100%) for SIB, 96.76% (range: 43.75%–100%) for self‐restraint, 95.91% (range: 25%–100%) for test‐item use, and 88.30% (range: 40.60%–100%) for toy engagement.

Procedural fidelity was assessed for 10.71% of trials for Nash, 14.10% for Jonah, 11.70% of trials for Evan, and 11.11% for Zuri. For each session, a data collector recorded whether (a) the protective procedures were arranged correctly (e.g., protective equipment was applied correctly) and session materials were present, (b) presession sampling was conducted with the test item and toy, (c) the therapist implemented the response promotion tactic correctly (e.g., prompted test‐item use after 10 s without test‐item use or delivered the edible according to the correct schedule), (d) the therapist implemented the response disruption tactic correctly (i.e., blocked or attempted to block the designated responses), and (e) the therapist returned any thrown test items the correct number of times. The number of correctly implemented components was divided by the total number of components, and the quotient was multiplied by 100. Mean fidelity was 99.64% (range: 97.83%–100%) for Nash, 99.83% (range: 85.30%–100%) for Jonah, 99.62% (range: 95.83%–100%) for Evan, and 100% for Zuri. Fidelity data are not available for Daniel because he did not complete the A‐CSA.

### Safeguards and protective procedures

Throughout the study, we employed several safeguards to reduce the risk of injury for all participants. Supporting Information F includes a summary of all individualized protections used in the functional analysis and A‐CSA for all participants; the checklist was adapted from Frank‐Crawford et al. (2024). Other protections included (a) obtaining institutional review board approval, (b) obtaining caregiver consent, (c) securing regular oversight by an external Data Safety Monitoring Board, (d) training all staff working with the participants to competency in behavior management skills, (e) implementing planned breaks from session if there were medical reasons participants could not fully participate, (f) consulting medical staff if sessions were terminated (i.e., the therapist ended the session before designated session duration elapsed due to the participant meeting individualized termination criteria), and (g) using abbreviated session durations. In total, the therapist terminated one session for Nash and three for Evan (the therapist did not terminate any sessions for Jonah or Zuri, and the A‐CSA was not completed with Daniel due to his avoidance of the test items during the presession probes). No participant incurred an injury during the A‐CSA.

### Procedure

#### Nomination and selection of alternative self‐restraint items for assessment

Potential alternative self‐restraint items included those that could be worn or through which individuals could voluntarily place their hands. The test items were selected for each participant based on a semistructured interview with each participant's primary clinician and in consideration of caregiver acceptability (determined via interviews between the primary clinician and caregiver). We sought to identify alternative self‐restraint test items that had the potential to successfully compete with the existing form of self‐restraint but not interfere with adaptive behavior. To that end, toys were available during all trials to provide opportunities for participants to engage in moderately preferred activities. The selected toys were identified from a previously conducted A‐CSA of leisure items.^2^ The toys were moderately preferred (engagement during the A‐CSA was between 50%–80%), but they did not compete with SIB or self‐restraint. It was important that the toys did not independently compete with SIB and self‐restraint so that we could isolate the effects of the potential competing self‐restraint test items on the outcome measures (self‐restraint, SIB, toy engagement). Table 3 lists the toys available in the A‐CSA trials.

**TABLE 3:** Trial duration, toys, and response promotion and disruption tactics.

P	Trial duration (min)	Toys	Response promotion tactic(s)	Response disruption tactic(s)
Nash	8	Stretchy worm, wand	PE^a^	RBRD (SIB)
			PE^a^	RBRD (SIB/SR)
Jonah	7; 2.50	Frog drum, popper	PE^a^	RBRD (SIB/SR)
			PE + NCR food^a^	RBRD (SIB/SR)
Evan	3	Leapfrog Scout toy	PE^a^	RBRD (SIB/SR)
Zuri	3	Music flower	‐	‐
Daniel	1	Moose, wand	‐	‐

*Note*: P = participant; PE = prompted engagement; RBRD = response blocking and redirection; SIB = self‐injurious behavior; SR = self‐restraint; NCR = noncontingent reinforcement.

^a^ Response promotion was conducted in conjunction with response disruption.

The alternative self‐restraint test items were classified into four general categories based on how they were to be used by the participant: (a) weighted test items that were worn (e.g., weighted vest), (b) protective clothing that was worn (e.g., hooded sweatshirt), (c) unweighted vests and pillows that were worn (e.g., airplane neck pillow), and (d) external test items into which the participant could insert their hands (e.g., a backpack with loops).

#### General procedures

##### Presession exposure probes

Prior to initiating the A‐CSA, participants were exposed to each alternative self‐restraint test item as the therapist modeled how they could be used or worn. The therapist then approached the participant and placed the test item on them or placed their hands and arms through or under the test item (for the loops and tabletop unit, respectively). The exposure probe was terminated if the participant avoided the test item (e.g., moved away from it, removed it, vocalized they did not like the test item, or displayed negative affect). Daniel avoided all test items during the presession exposure probes, so he did not complete the remainder of the A‐CSA.

##### Trial duration

Trial duration was individualized to maximize efficiency, decrease risk to participants with higher rates of SIB, and ensure the duration was sufficient to allow adequate opportunity to observe target behavior for participants with lower rates of SIB (for additional details, see Hagopian et al., 2020). Trial duration was calculated by dividing 10 by the mean rate of SIB in the no‐interaction condition of the functional analysis. For both, the calculated value was rounded up or down to the closest whole minute (the only exception to this was for Jonah for whom we reduced the session duration from 7 min to 2.50 min in conditions where food was provided as a reinforcer to limit the potential for satiation). Table 3 includes the individual trial durations, which ranged 1–8 min.

##### General progression of conditions

The A‐CSA involved multiple conditions, each of which are briefly summarized here, then described in further detail below. After presession exposure probes, the assessment proper was initiated. The A‐CSA included a no‐item control trial in which only the toys were available and test trials that included one of the alternative self‐restraint test items along with the toys. Thus, the test and control trials were identical other than the presence of the alternative self‐restraint test items. A series included one control trial and one test trial for each test item assessed. The control trial was always conducted first in the series, followed by the test trials in a quasirandomized order. For each participant, we completed three series and averaged outcomes across the series.

**FIGURE 1:**
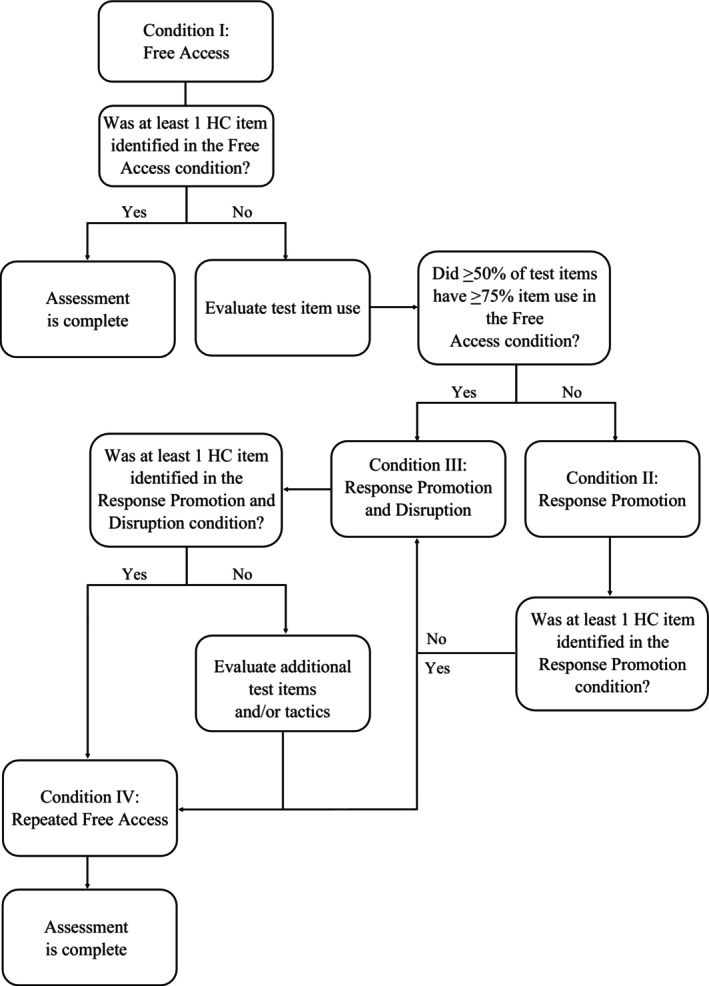
Flowchart for progression through conditions. The repeated free‐access condition was only conducted with Jonah, as this condition had not been added to the protocol at the time of participation for Nash and was unnecessary to conduct with Evan and Zuri because a high‐competition (HC) item was identified in a free‐access condition in the absence of promotion or disruption tactics. The flowchart depicts progression through the assessment based on responding in each condition; participants did not experience each condition if they did not meet criteria.

In the test trials of all conditions, test items were placed on the individual or their hands were placed into the test item at the beginning of the trial. In the first condition (free access), test items could be freely used or removed (i.e., an individual could remove the test item placed on them at the start of the session). If at least one HC item was not identified in the free‐access condition, then additional conditions were conducted during which various augmenting tactics were employed. This included a response promotion condition during which tactics such as prompting test‐item use were employed, and a response promotion and disruption condition in which tactics such as prompting and response blocking were used together to concurrently limit SIB or self‐restraint and promote test‐item use. In the final repeated free‐access condition, all augmenting tactics were withdrawn to determine whether improved outcomes persisted after contacting the consequences associated with increased test‐item use. Figure 1 includes a flowchart depicting the progression through conditions of the A‐CSA. Note however, that not all conditions were experienced by each participant.

#### Conditions

##### Condition I: Initial free access

The purpose of this condition was to evaluate the effects of access to each test item on SIB and self‐restraint (as well as engagement with toys) in the absence of other manipulations. During both test and control trials, no consequences were delivered for test‐item use or toy engagement. All SIB and self‐restraint were ignored (or blocked for safety reasons and then ignored, as specified in Supporting Information F). During the control trial, the toys were placed on the table in front of the participant, and the participant was informed they could engage with them if they wanted (no test items were present). During test trials, test items were placed on the individual or their hands were placed through the test item at the start of the trial; thereafter, the participant could freely use the test item (i.e., they could remove or cease engaging with the test item at any time). If the participant threw the test item, the therapist waited 5 s and then re‐presented the test item on the table once. If any additional disruptions occurred, they were ignored, and the test item was left where it landed (but the participant could independently retrieve the test item). The same contingencies were in place for disruptions with the toys. If at least one HC item was identified in the free‐access condition, the assessment was considered complete.

*Individualization*. For Evan, a second free‐access condition was conducted after it was observed that contingent application of a weighted hat in the initial free‐access condition reduced but did not completely suppress his SIB. The second free‐access condition was identical to the initial free‐access with the exception that he wore the weighted hat noncontingently (throughout the entire trial) during all control and test trials rather than it being applied contingently. For Zuri, who was legally blind, the test items were placed on her while explaining how it was going to be applied. If she resisted the application of the test item, then it was left within arm's reach.

##### Condition II: Response promotion

The purpose of this condition was to promote the use of the test items as a competing response for self‐restraint and SIB while maintaining or increasing toy engagement. No participant met criteria to experience the response promotion condition because test‐item use was high across all participants in the free‐access condition. However, it would have been conducted with participants if the free‐access condition (a) had failed to identify at least one HC item and (b) if test‐item use was below 75% for more than 50% of all tested items. If participants had met criteria for this condition, the following procedures would have been conducted: If 10 s had elapsed without test‐item use, then the therapist would have physically guided the participant to use the test item for 5 s while vocally stating how the test item could be used (e.g., “You can wear the blanket by wrapping it around your shoulders”). The same prompting of toy engagement would have occurred if 10 s elapsed without toy engagement. If 10 s elapsed without test‐item use or toy engagement, we would have prompted use of the test item first and then engagement with the toys.^3^ Prompted engagement with test items or toys would have been terminated for 10 s if the participant exhibited avoidance.

##### Condition III: Response promotion and disruption

The response promotion and disruption condition was conducted with participants if (a) the individual had high levels of test‐item use during free access but no HC items were identified (this occurred with Nash and Jonah) or (b) the response promotion condition was conducted but no HC items were identified (this did not occur for any participant). All SIB or attempts to engage in self‐restraint were blocked and followed by prompted engagement (redirecting the participant to engage with the test item, the toy, or both as described in the prompted engagement condition). Independent of the occurrence of SIB and self‐restraint, prompted engagement was still implemented following 10 s without test‐item use or toy engagement. Prompted engaged with test items or toys was terminated for 10 s if the participant exhibited avoidance.

*Individualization*. The response disruption procedures for Nash and Jonah were response blocking and redirection. For Nash, two different response promotion and disruption conditions were conducted. In the first response promotion and disruption condition, only topographies of SIB were blocked and redirected (i.e., neck‐directed SIB and head‐banging). Because rates of SIB persisted and no HC items were identified under these contingencies, a second response promotion and disruption condition was conducted in which both SIB and self‐restraint were blocked and redirected. The procedures were otherwise identical to Nash's first response promotion and disruption condition.

In the second response promotion and disruption condition for Jonah, noncontingent food (identified from a paired stimulus preference assessment; Fisher et al., 1992) was provided to increase background reinforcement. The therapist alternated delivering two preferred foods; a small piece of food was delivered noncontingently at the start of session and then additionally throughout session as soon as the therapist believed Jonah had finished consuming the previous piece. To isolate any effects that the test items had on SIB and self‐restraint and adaptive behavior rather than the effects of reinforcement (i.e., food and toys), both the food and toys were also present in the control condition. After conducting a few sessions with food, trial duration was decreased from 7 min to 2.50 min to limit the number of calories Jonah consumed during sessions and avoid possible confounds associated with satiation.

##### Condition IV: Repeated free access

The repeated free‐access condition was identical to the initial free‐access condition. That is, the promotion (prompted engagement and noncontingent food) and disruption (response blocking and redirection) tactics were removed. Repeated free access was conducted only after HC items were identified in the response promotion or response promotion and disruption conditions. Therefore, this condition was not conducted if (a) HC items were identified during initial free access (in which case the assessment was complete) or (b) the response promotion or response promotion and disruption conditions were conducted without identifying a HC item. The primary purpose of this condition was to determine whether improved outcomes observed during response promotion or response promotion and disruption conditions persisted without the additional procedures included in those conditions, suggesting competing self‐restraint responses had been established. The repeated free‐access condition was only conducted with Jonah, as this condition had not been added to the protocol at the time of Nash's participation and was unnecessary to conduct with Evan or Zuri because an HC item was identified in one of the initial free‐ access conditions.

## RESULTS

### Individual participant results

**FIGURE 2:**
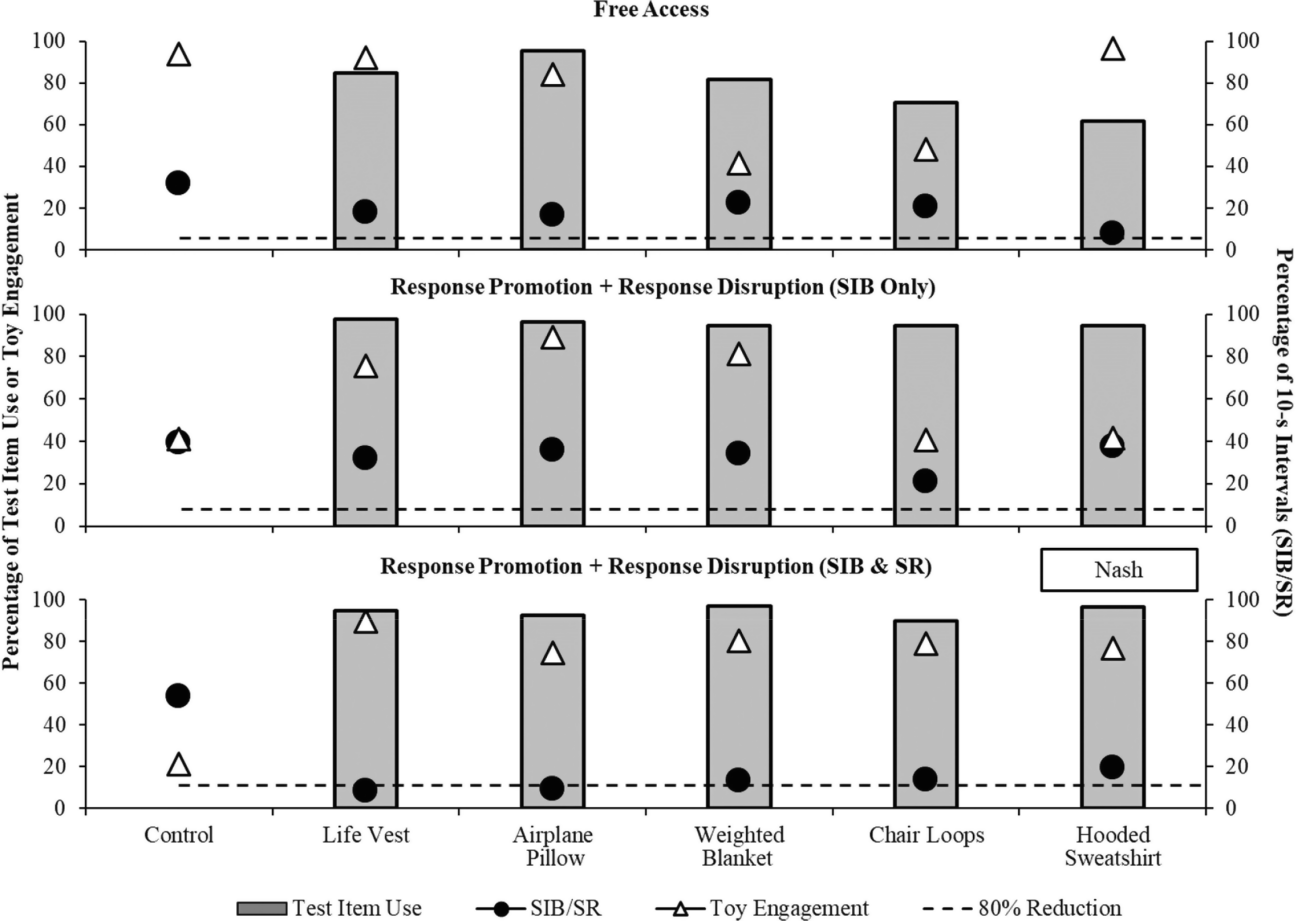
Outcomes for Nash. The dashed horizontal line denotes an 80% reduction from the respective control. SIB = self‐injurious behavior; SR = self‐restraint.

**FIGURE 3:**
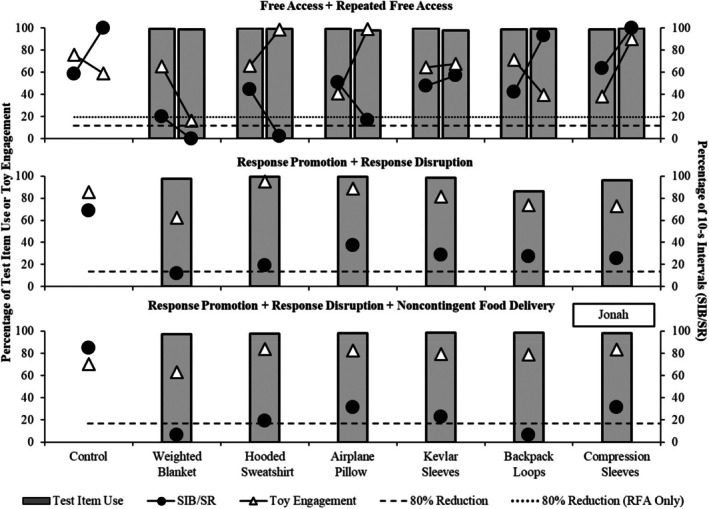
Outcomes for Jonah. The dashed horizontal line denotes an 80% reduction from the respective control. The dotted horizontal line denotes an 80% reduction from the repeated free‐access condition. SIB = self‐injurious behavior; SR = self‐restraint; RFA = repeated free access.

**FIGURE 4:**
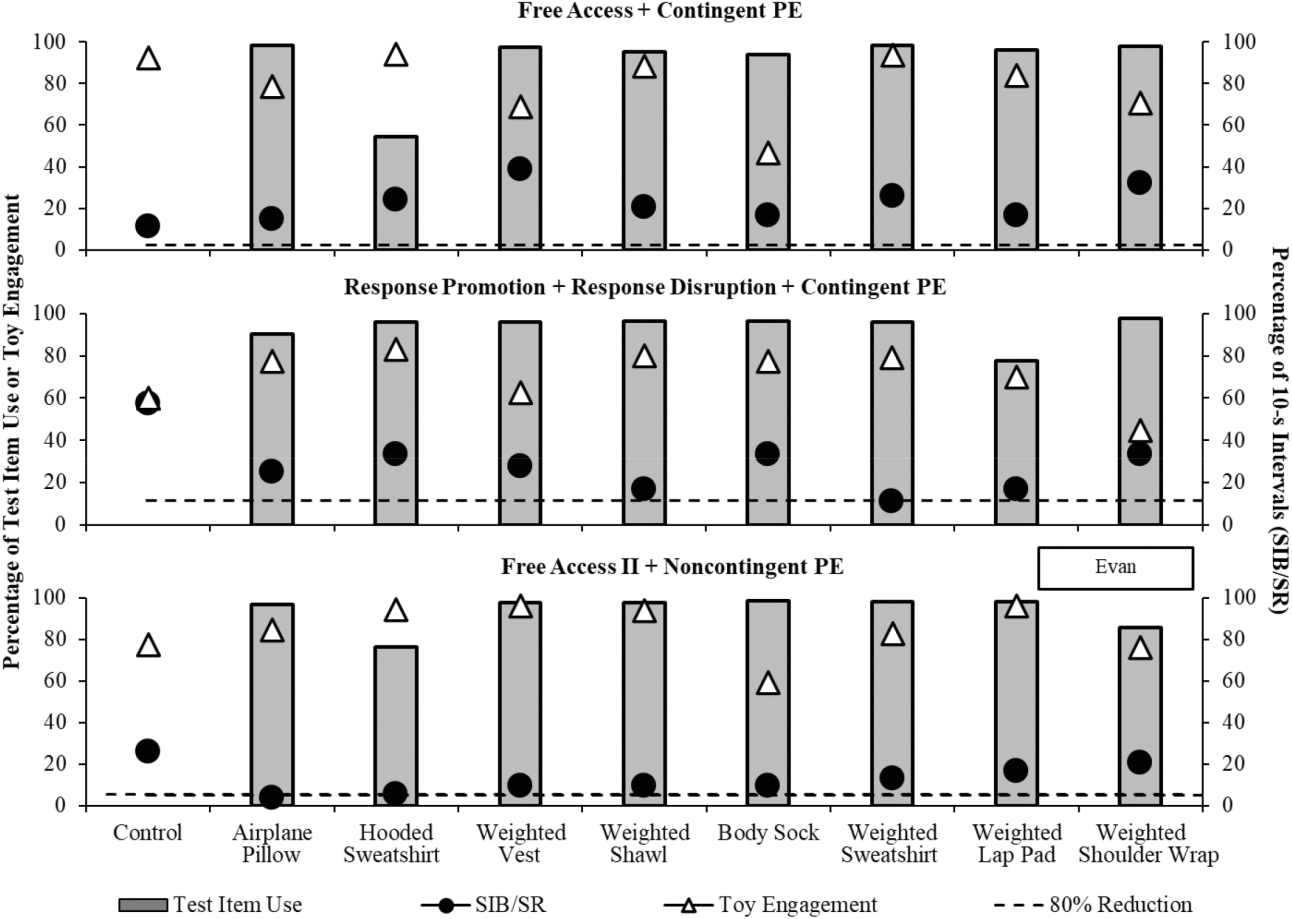
Outcomes for Evan. The dashed horizontal line denotes an 80% reduction from the respective control. PE = protective equipment; SIB = self‐injurious behavior; SR = self‐restraint.

**FIGURE 5:**
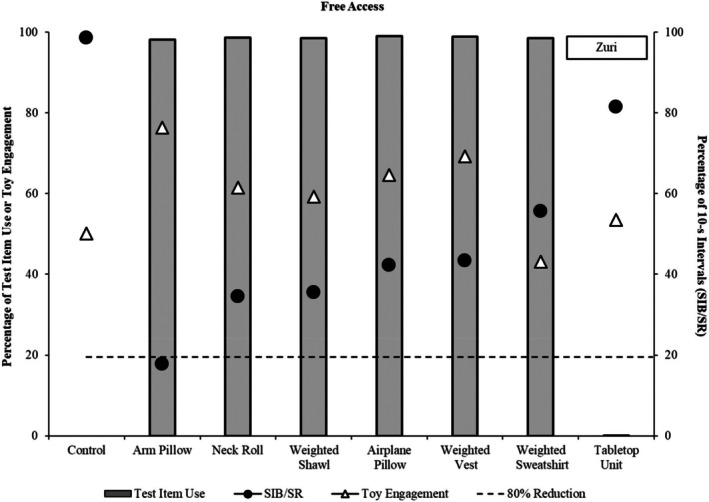
Outcomes for Zuri. The dashed horizontal line denotes an 80% reduction from the control. SIB = self‐injurious behavior; SR = self‐restraint.

Figures 2, 3, 4, and 5 depict outcomes from Nash, Jonah, Evan, and Zuri, respectively. Each condition is depicted in a separate panel in the order it was experienced. For Jonah, the free‐access and repeated free‐access conditions are depicted juxtaposed in the top panel to observe the changes in outcomes before and after exposure to the other conditions.

For Nash (see Figure 2), no HC items were identified in the free‐access condition, although the hooded sweatshirt was associated with a 75.88% reduction in the percentage of intervals with SIB and self‐restraint relative to the control trial. When only SIB was blocked in the first response promotion and disruption condition, SIB and self‐restraint did not decrease for any test item. Toy engagement was variable across test items in the free‐access and first response promotion and disruption conditions. Because no HC item was identified, a second response promotion and disruption condition was conducted during which both SIB and self‐restraint were blocked. During this condition, we identified two items associated with at least an 80% reduction in the percentage of intervals with SIB and self‐restraint relative to the control trial, a child‐sized life vest and airplane pillow. Importantly, toy engagement was high and increased relative to the control trial for the two items, indicating that use of these items did not interfere with appropriate toy engagement. Thus, the child‐sized life vest and airplane pillow met criteria for classification as HC items. As noted, the repeated free‐access condition was not yet part of the protocol at the time of Nash's enrollment, so we cannot conclude whether the use of these items facilitated an alternative self‐restraint response independent of the response promotion and disruption tactics.

For Jonah (see Figure 3), although test‐item use was high across all test items, no HC items were identified in the free‐access condition. The bottom two panels depict the two response promotion and disruption conditions. Test‐item use remained high, and the weighted blanket was associated with an 80% reduction in SIB and self‐restraint relative to the control trial during the initial response promotion and disruption condition; however, toy engagement decreased by 23.28% relative to the control trial. Because no HC items were identified, we conducted a second response promotion and disruption condition with the addition of food reinforcement. We identified two HC items (weighted blanket and backpack loops); with both items, the percentage of intervals with SIB and self‐restraint decreased by more than 80% relative to the control trial and toy engagement remained high. When the promotion and disruption tactics were withdrawn in the repeated free‐access condition, item use remained high and two items were identified as HC items: the hooded sweatshirt and airplane pillow. Although the weighted blanket was associated with an 80% reduction in SIB and self‐restraint, toy engagement decreased relative to the control trial—the weighted blanket was therefore not considered an HC item.

We did not identify any HC items for Evan (see Figure 4) in the first free‐access condition. The middle panel depicts outcomes of the response promotion and disruption condition during which Evan was redirected to engage with the toy following head‐directed SIB (which was always blocked for safety reasons). Although the weighted sweatshirt was associated with an 80% reduction in SIB and self‐restraint relative to the control trial, thereby meeting the definition of a HC item, the percentage of intervals with SIB and self‐restraint (*M =* 11.11%) were similar to those observed in the first free‐access condition (*M =* 25.92%). We felt that this decrease was not necessarily clinically significant and decided to modify the A‐CSA further in pursuit of larger reductions in SIB and self‐restraint. Interestingly, we observed that when the weighted hat was applied contingently for safety reasons (following the tenth occurrence of head‐directed SIB), SIB often decreased. Thus, rather than adding other promotion or disruption tactics to further reduce SIB and self‐restraint, we elected to instead conduct a second free‐access condition using the weighted hat as protective equipment worn noncontingently throughout all trials. We felt that this would be less intrusive than blocking and physical redirection, and his parents reported they found the hat more socially acceptable than a padded helmet. In the second free‐access condition, SIB and self‐restraint occurred at overall lower levels across most test items (and remained relatively unchanged in the control trial). We identified one HC item (the airplane pillow), and a second item, the hooded sweatshirt, was associated with a 78.51% decrease relative to the no‐item control trial. Toy engagement remained high with all test items except the body sock. The repeated free‐access condition was not conducted given that we identified at least one HC item in a free‐access condition.

Finally, for Zuri (see Figure 5), one test item was identified as an HC item in the free‐access condition, the arm pillow. This item was associated with at least an 80% reduction in SIB and self‐restraint as well as marked increases in toy engagement, both relative to the control trial. Thus, augmenting tactics were not evaluated.

### Summative results

**FIGURE 6:**
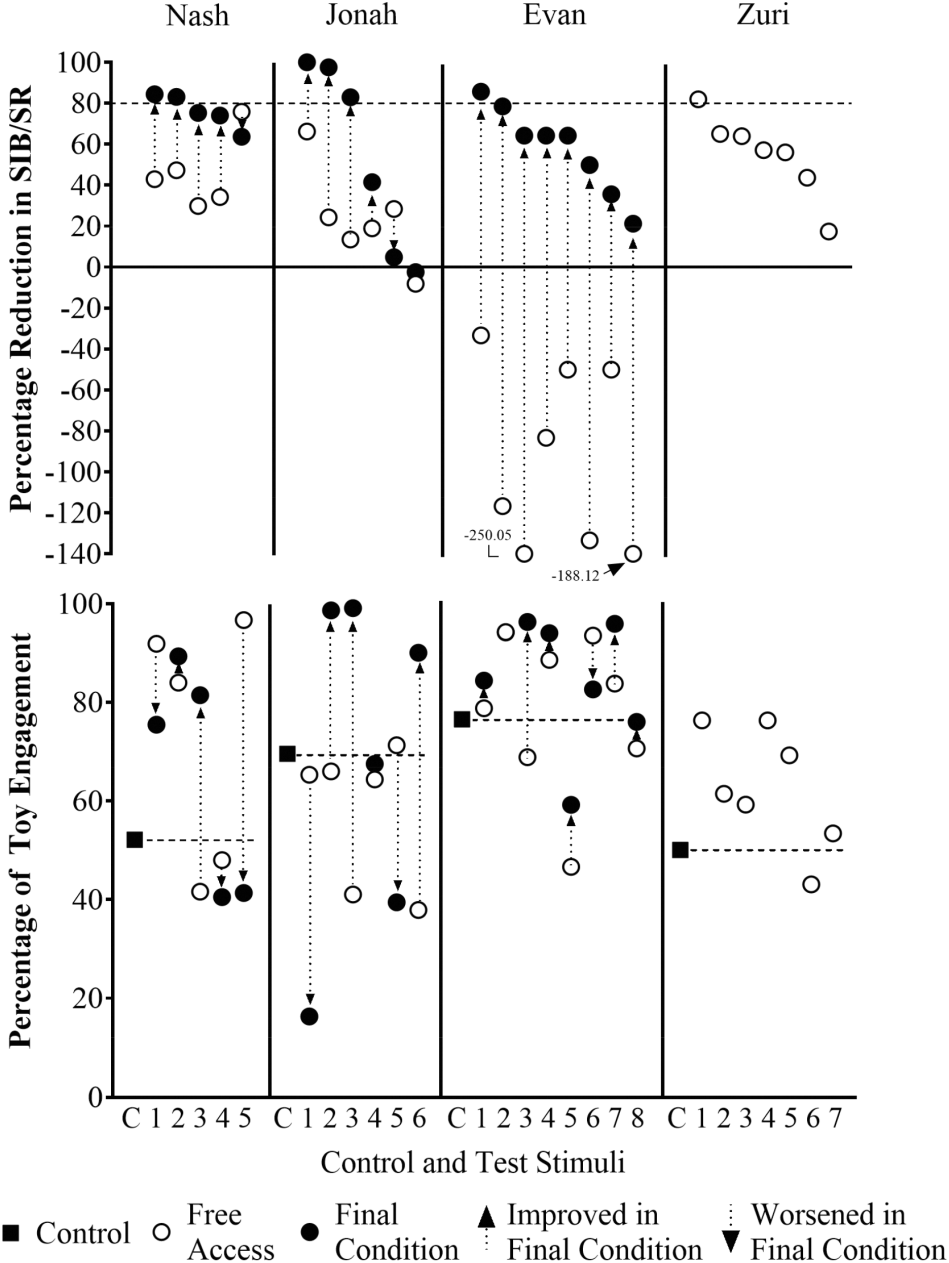
Percentage of change in 10‐s intervals with SIB and self‐restraint and percentage of toy engagement across first and final condition. The control and each test item is represented by a C (control) or number (test item) displayed below the panels, outcomes for test items are depicted as a set of connecting data points, and each condition is denoted by a different symbol. The dashed horizontal line represents an 80% reduction in challenging behavior (top panel) or the mean percentage of toy engagement that occurred across all control trials (bottom panel) for each participant. SIB = self‐injurious behavior; SR = self‐restraint.

Table 4 lists the number of HC items identified in each condition of the A‐CSA for each participant. The top panel of Figure 6 depicts the percentage of change in SIB and self‐restraint for each test item relative to the control trial across the first and final condition of the A‐CSA for all participants. Points above the dashed line represent test items that were associated with an 80% or greater reduction relative to the control trial. The bottom panel depicts the percentage of session with toy engagement while each test item was available; the percentage of session with toy engagement during the control trial is depicted by the closed square aligned with the horizontal dashed line. The final condition was response promotion and disruption during which both SIB and self‐restraint were blocked for Nash, repeated free access for Jonah, and the second free‐access condition for Evan. No final condition is depicted for Zuri because only the free‐access condition was conducted. Many of the test items resulted in some level of reduction in the percentage of intervals with SIB and self‐restraint relative to the control trial. Specifically, of the 26 items tested, 17 (65.38%) resulted in a reduction in the initial free‐access condition. Notably, all eight items tested resulted in an increase in SIB and self‐restraint for Evan. The final condition for Nash (response promotion and disruption), Jonah (repeated free access), and Evan (second free access) resulted in a greater percentage of reduction in SIB and self‐restraint than did the initial free‐access condition for 16 of the 19 test items (84.21%). No substantial change was observed for two items (10.53%), and a relatively larger percentage reduction was observed in free access for one item (5.26%). In total, 23.08% of the items tested (*n =* 6) were ultimately identified as HC items.

**TABLE 4:** Number of high‐competition items identified.

P	# of Test Items	Number of high‐competition items identified			
		Free access	Response promotion	Response promotion and disruption	Repeated free access
Nash	5	0	‐	0; 2	‐
Jonah	6	0	‐	1; 2	2
Evan	8	0; 1	‐	1	‐
Zuri	7	1	‐	‐	‐

*Note*: The semicolon separates the number of high‐competition items identified across the two response promotion and disruption conditions (Nash and Jonah) or two free‐access conditions (Evan). P = participant. For Daniel, results are not depicted because he avoided the test items during presession exposure.

Outcomes were more mixed with respect to changes in toy engagement across the first and final conditions (see Figure 6, bottom panel). During free access, Nash engaged with the toys more often with three of the test items relative to the control trial (Items 1, 2, and 5); toy engagement was similar to the control for Test Items 3 and 4. During the final condition, toy engagement remained elevated relative to the control trial for both HC items (Test Items 1 and 2); however, toy engagement decreased modestly relative to the free‐access condition for Test Item 1. For Jonah, toy engagement was similar to the control trial for four of the six test items (Test Items 1, 2, 4, and 5) during free access and decreased for two items (Test Items 3 and 6). During the final condition, engagement with the toys increased relative to the control trial and to levels observed in free access for three items (Test Items 2, 3, and 6), two of which were HC items (Test Items 2 and 3). Toy engagement decreased relative to the control trial and free access for Test Item 1. In Evan's first free‐access condition, toy engagement increased relative to the control trial for Test Items 2, 4, 6, and 7; stayed about the same for Test Item 1; and decreased for Test Items 3, 5, and 8. During the second free‐access condition, engagement increased relative to the control trial for six items (Test Items 1, 2, 3, 4, 6, and 7), stayed about the same for Test Item 8, and decreased for Test Item 5. Finally, for Zuri, toy engagement was relatively equal to or greater than levels observed in the control trial for all seven test items, and toy engagement increased with the HC item (Test Item 1) relative to levels observed in the control trial.

## DISCUSSION

The A‐CSA was adapted for individuals who exhibited automatically maintained SIB and self‐restraint (classified as Subtype 3). It was designed to identify alternative, competing self‐restraint items that did not interfere with adaptive behavior and could replace the individual's existing form of self‐restraint without increasing SIB. At least one HC item was identified for each of the four participants who completed the A‐CSA.

The current study aligns with prior research on the A‐CSA in that augmenting tactics were sometimes necessary to identify competing stimuli. Frank‐Crawford et al. (2023) and Hagopian et al. (2020) included six participants with Subtype 3 SIB. Augmenting tactics were necessary to identify competing leisure stimuli for four of those participants. In the current study, we also found it necessary to include response promotion and disruption tactics for three participants—Nash, Jonah, and Evan. However, the current study is limited in that the repeated free‐access condition, which informs whether the augmenting tactics produced lasting change after they are withdrawn, was only evaluated with Jonah. We did not conduct the repeated free‐access condition with Nash because it was not part of the procedure at the time we conducted the A‐CSA with him. For Evan, response promotion and disruption tactics were evaluated but deemed unnecessary after a protective procedure was modified in the second free‐access condition. Thus, for Nash and Evan, the independent effects of the test items are unknown. When the repeated free‐access condition is not conducted or fails to identify HC items, practitioners may consider including augmenting tactics in their treatment evaluations (e.g., Jennett, Jann, & Hagopian, 2011; Leif et al., 2020). The use of such tactics is not ideal because of the increased response effort and continuous monitoring required for prompting and blocking, but they could possibly be faded over time to establish independent use of the HC items. Future studies should include a repeated free‐access condition to examine whether augmenting tactics could be removed or must be faded later.

The current study extends Rooker and Roscoe (2005) by examining the extent to which the test items compete with existing forms of self‐restraint while maintaining low levels of SIB and without decreasing toy engagement. Although we identified six items that met these criteria, we also observed that the weighted blanket reduced Jonah's self‐restraint and SIB while substantially reducing his toy engagement. Thus, we did not consider this item to be an appropriate alternative to his current form of self‐restraint. It is possible that toy engagement could increase over time as continued reductions in self‐restraint and SIB allow the individual to contact reinforcement for engagement with toys. This represents an important avenue for future research.

Relatedly, we did not define an HC item based on the degree to which a participant used the test item or engaged with the toys for two reasons. First, test‐item use was almost universally high, likely because we excluded any item that the participant avoided during presession exposure. However, one limitation is that we did not assess the level of difficulty associated with removing test items. It is possible some were difficult to remove, which may have inflated measures of test‐item use. Second, prior research on CSAs has shown that high levels of toy engagement (i.e., preference) do not predict the extent to which stimuli reduce challenging behavior (i.e., competition; Frank‐Crawford et al., 2023; Haddock & Hagopian, 2020; Laureano et al., 2023). The same is true in the current study. There were minimal differences in toy engagement between (a) test items associated with an 80% reduction and (b) those associated with less than an 80% reduction (77% vs. 72.76% of session with toy engagement, respectively).

We only measured a single form of adaptive behavior—toy engagement. It is conceivable that one would use HC items across different situations, including demand contexts. Thus, future researchers should include multiple measures of adaptive behavior to determine whether the same HC item can be used across contexts or if different HC items are needed. The toys included in the current A‐CSA were selected because they were preferred but did not function as competing stimuli. It is likely that in practice, competing self‐restraint items would be used in combination with other, established competing toys (e.g., Hagopian et al., 2020) or tasks (e.g., Schmidt et al., 2021). Thus, future researchers should also examine the interactive effects of competing self‐restraint items, toys, and tasks.

Our understanding of automatically maintained SIB is inherently limited by the fact that we cannot directly observe or control the reinforcers that occasion and maintain it (Vollmer, 1994). However, we can make inferences about its controlling variables by examining how SIB changes across conditions and how it covaries with other behaviors. When self‐restraint co‐occurs with SIB, we can infer that SIB produces aversive consequences, which evoke self‐restraint because it may have historically impeded SIB (see Fisher & Iwata, 1996). Self‐restraint was selected as a defining feature for Subtype 3 by Hagopian et al. (2015) based on this hypothesized avoidance mechanism.

Some individuals engage in self‐restraint almost continuously, whereas others vacillate between SIB and self‐restraint. Vacillations between self‐restraint and SIB may be the product of changes in the relative strength of the mechanisms for each response. When self‐restraint predominates, this could suggest its establishing operation is relatively strong (e.g., aversive consequences of SIB are more potent). When SIB predominates, this could suggest that the mechanisms that underlie SIB may be at strength (e.g., the reinforcing consequences are more potent) or it could highlight possible mechanisms related to motor dysfunction (Hagopian & Frank‐Crawford, 2018). Although the current procedures do not allow examination of these mechanisms, the vacillation between responses is not uncommon and highlights the need for research to understand why this occurs.

One possible mechanism that could explain the vacillation between SIB and self‐restraint is related to changes in pain sensation to traumatized tissue over time. This hypothesis is informed by research on the pathophysiology of pain, which indicates that physical trauma induces immediate pain and increases pain sensitivity over time (i.e., *delayed hyperalgesia*; Schaible & Richter, 2004). Another delayed effect of physical trauma is *allodynia*: the perception of pain from nontraumatic sensory stimulation like light touching to a previously traumatized area (Sandkühler, 2009). These delayed increases in sensitivity to pain and other stimulation may be biologically protective—minimizing contact to traumatized area and avoiding additional tissue damage. The extent to which delayed hyperalgesia and allodynia are mechanistically involved in self‐restraint is not known but could be studied by examining if nontraumatic sensory stimulation to an area traumatized by SIB produces different reactions depending on whether SIB or self‐restraint is the predominant response. If light touching evokes more avoidance during periods when self‐restraint predominates, this could support the hypothesis that self‐restraint is occurring to avoid aversive sensory consequences of SIB. In addition to measuring withdrawal, changes in facial expressions and autonomic arousal (e.g., heart rate variability galvanic skin response) could be obtained to determine whether reactivity to stimulation is accentuated when self‐restraint predominates (Defrin et al., 2021; Gunderson et al., 2025).

Although these initial findings are generally positive, additional research is needed to determine whether competing self‐restraint items produce clinically meaningful benefits. Future studies must examine whether use of these items is more socially acceptable, less debilitating, reduces SIB more, or reduces risk of injury compared to existing forms of self‐restraint. Another intriguing area for future research is whether establishing a novel competing response for SIB has application beyond replacing maladaptive self‐restraint. SIB classified as Subtype 2 is also highly treatment resistant, and individuals with Subtype 2 incur the most severe injuries (Rooker et al., 2020). However, those with Subtype 2 do not engage in self‐restraint. Considering the severity of SIB classified as Subtype 2, teaching a novel and adaptive competing response for SIB could be further explored. This aligns with a function‐based approach to treatment in which interventions are designed based on an understanding of the controlling variables (e.g., functional communication training; Carr & Durand, 1985). To the extent that socially acceptable alternative self‐restraint items are identified, voluntarily used, and result in reduced risk of harm without interfering with adaptive functioning, use of these items may be considered a form of self‐control. Including such measures in future research would be noteworthy.

The current study is also limited because we did not include an intervention phase to further evaluate the HC items. This represents an important area for future research to (a) validate the outcomes of the A‐CSA and (b) evaluate the durability of the HC items. Interestingly, test items associated with an 80% reduction in self‐restraint and SIB sometimes varied across phases. Thus, additional research is needed to examine the durability of these items over time. In addition, the competing self‐restraint items are not intended to be used alone; thus, future research should consider conducting several A‐CSAs to also identify competing toys (Hagopian et al., 2020) and tasks (Schmidt et al., 2021). When arranged together, these stimuli may improve treatment outcomes by increasing the amount and varying the sources of response and reinforcer competition for SIB and self‐restraint.

Finally, we made several procedural modifications for individual participants. Although the outcomes were positive, knowing when and what type of modifications to make may require a certain level of expertise in SIB, self‐restraint, and A‐CSAs that, currently, not every clinician will have. However, as the A‐CSA protocol is implemented with more individuals who exhibit Subtype 3 automatically maintained SIB, patterns may emerge to help inform such decision making.

Research on the treatment of self‐restraint has been quite limited in recent years. Although only initial outcomes are reported in the current study, it may contribute to the literature on self‐restraint by suggesting some possible mechanisms that could explain how self‐restraint functions as an avoidance response. Furthermore, the current study expands prior research on alternative self‐restraint items (Rooker & Roscoe, 2005) and the A‐CSA (Hagopian et al., 2020) by advancing an approach to treatment focused on identifying or establishing competing items to replace existing forms of self‐restraint.

## AUTHOR CONTRIBUTIONS

MFC contributed to conceptualization, methodology, project administration, supervision, and writing. LH contributed to conceptualization, funding acquisition, methodology, supervision, and writing. JS contributed to conceptualization, project administration, and supervision. GR contributed to conceptualization, methodology, and project administration. DP contributed to data curation, investigation, visualization, and writing. RB contributed to data curation and investigation.

## CONFLICT OF INTEREST STATEMENT

The authors have no conflicts of interest to declare.

## ETHICS APPROVAL

The study received approval from an institutional review board, and the participants' caregivers provided consent for participation.

## Supporting information


**Data S1:** Supporting Information

**Data S2:** Supporting Information

## Data Availability

Supporting Information A includes additional details on the types of alternative self‐restraint items; Supporting Information B, C, D, and E include additional figures with self‐injury and self‐restraint data separated for Nash, Jonah, Evan, and Zuri, respectively; and Supporting Information F includes a checklist of safety measures employed during the study. Additional reasonable requests for data can be directed to the corresponding author.

## Footnotes

^1^
Findings from some studies suggest self‐restraint can be positively reinforcing (e.g., Rooker & Roscoe, 2005) and maintained by social reinforcement (Derby et al., 1996).

^2^
Outcomes from the A‐CSA for toys are described in Frank‐Crawford et al. (2023) for Daniel (P13), Nash (P14), Jonah (P15), and Zuri (P 16). Data from Evan's A‐CSA are available on request.

^3^
For ease of description, we will refer to prompting test‐item use as prompted engagement even though we otherwise refer to interaction with the test items as “use” rather than “engagement.”
