# Supplementary material for: Application of the augmented competing stimulus assessment to identify and establish competing self‐restraint items
Source: J Appl Behav Anal. 2025 Nov 3;59(1):e70040. doi: 10.1002/jaba.70040 (PMC12689246; doi:10.1002/jaba.70040)

Supporting Information

Supporting Information A

Description of Test Items and Respective Operational Definitions for Use

Category	SCI	Description	Operational Definition for Use
Weighted items	Weighted blanket	A soft blanket with distributed weight that is wrapped around the shoulders	Use occurred if the participant kept at least one body part underneath or wrapped in the blanket or gripped it with the hands
	Weighted scarf	A scarf with weight distributed evenly that is placed freely around the neck	Use occurred if the participant kept the scarf on their neck or over any other part of the body
	Weighted shawl	A soft wrap with weights distributed along the edge that is placed around the shoulders	Use occurred if the participant kept a least one body part underneath or wrapped up in the shawl or gripped it with the hands
	Weighted shoulder wrap	A shoulder wrap with weight distributed evenly that is worn over the shoulders and secured on the chest with a snap closure	Use occurred if the participant kept the wrap on their shoulders, regardless of whether the snap was secured, or any part of the body
	Weighted sweatshirt	A zip-up sweatshirt with weight distributed long the zipper and hood	Use occurred if the participant had the weighted sweatshirt on their body, zipped or unzipped with the hood up or down
	Weighted vest	A vest with weight distributed along the front and back that is placed over the head and secured around the torso with Velcro	Use occurred if the participant kept the vest on their body, regardless of whether or not the Velcro was attached
	Wrist weights	A pair of straps with a variable amount of weight inside secured around the wrists with Velcro	Use occurred if the participant kept the wrist weights attached securely on one or both wrists
Protective clothing	Compression sleeves	A pair of tight, stretchy sleeves worn on the arms over the elbow	Use occurred if the participant wore the sleeves on one or both arms
	Hooded sweatshirt	A hooded sweatshirt with two vertical cuts on the front pocket	Use occurred if the participant had hand(s) inserted through one or both of the slits on the pocket

	Kevlar sleeves	A pair of soft, thick sleeves worn on the arms over the elbow	Use occurred if the participant wore the sleeves on one or both arms
	Quarterback pocket	A soft pouch worn across the waist and secured around the torso with a strap and buckle	Use occurred if the participant put one or both hands through the openings on each side of the quarterback pocket
Non-weighted pillows and vests	Airplane pillow	A soft, padded pillow worn around the neck	Use occurred if the participant kept the airplane pillow around the neck
	Arm pillow	A fluffy pillow with an extended arm to wrap around oneself	Use occurred if the pillow was worn around the neck, placed on the body, or gripped with the hands
	Life vest	A flotation device worn over the head and attached around the torso with buckles	Use occurred if the participant kept the life vest on the body, with or without the straps buckled
	Neck roll	A soft padded roll worn around the neck and securely tied behind the back	Use occurred if the neck roll was worn around the neck
External self-control items	Backpack loops	A pair of soft moleskin wrist straps that are attached to a backpack with carabineers	Use occurred if the participant kept one or both arms secured in the loop
	Body loops	A single soft moleskin wrist strap that the participant sits on top of and is secured around both wrists using Velcro	Use occurred if the participant kept one or both arms secured in the loops
	Body sock	A large piece of stretchy fabric with an opening down the middle that is worn by placing the entire body inside the opening	Use occurred if the participant kept at least one arm or leg within the body sock or wrapped up in the fabric
	Chair loops	A pair of soft moleskin wrist straps that are attached to a chair with Velcro	Use occurred if the participant kept one or both arms secured in the loops
	Lap pad	A flat rectangular pad with weight distributed evenly that is placed on top of the lap	Use occurred if the participant kept the pad on their lap or draped over another body part
	Neck massager	A long, soft tube producing light vibration that is placed freely around the neck	Use occurred if the participant kept the massager around the neck or over any other body part

Tabletop unit	A box made of wood and clear Plexiglas with one open side to insert the hands/arms	Use occurred if the participant kept hands underneath the tabletop unit

Supporting Information B

Nash's Outcomes Separated by Self-Restraint and Self-Injurious Behavior

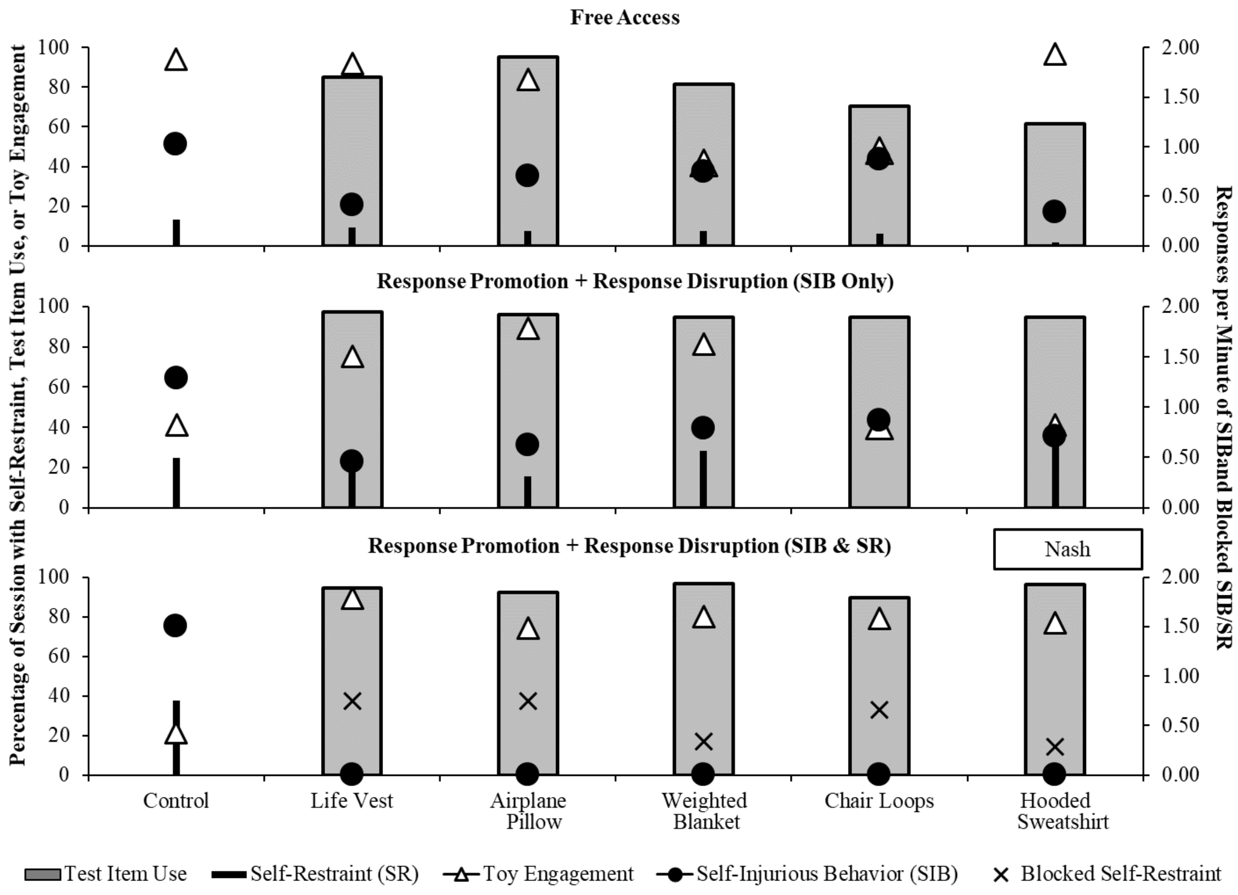

Supporting Information C

Jonah's Outcomes Separated by Self-Restraint and Self-Injurious Behavior

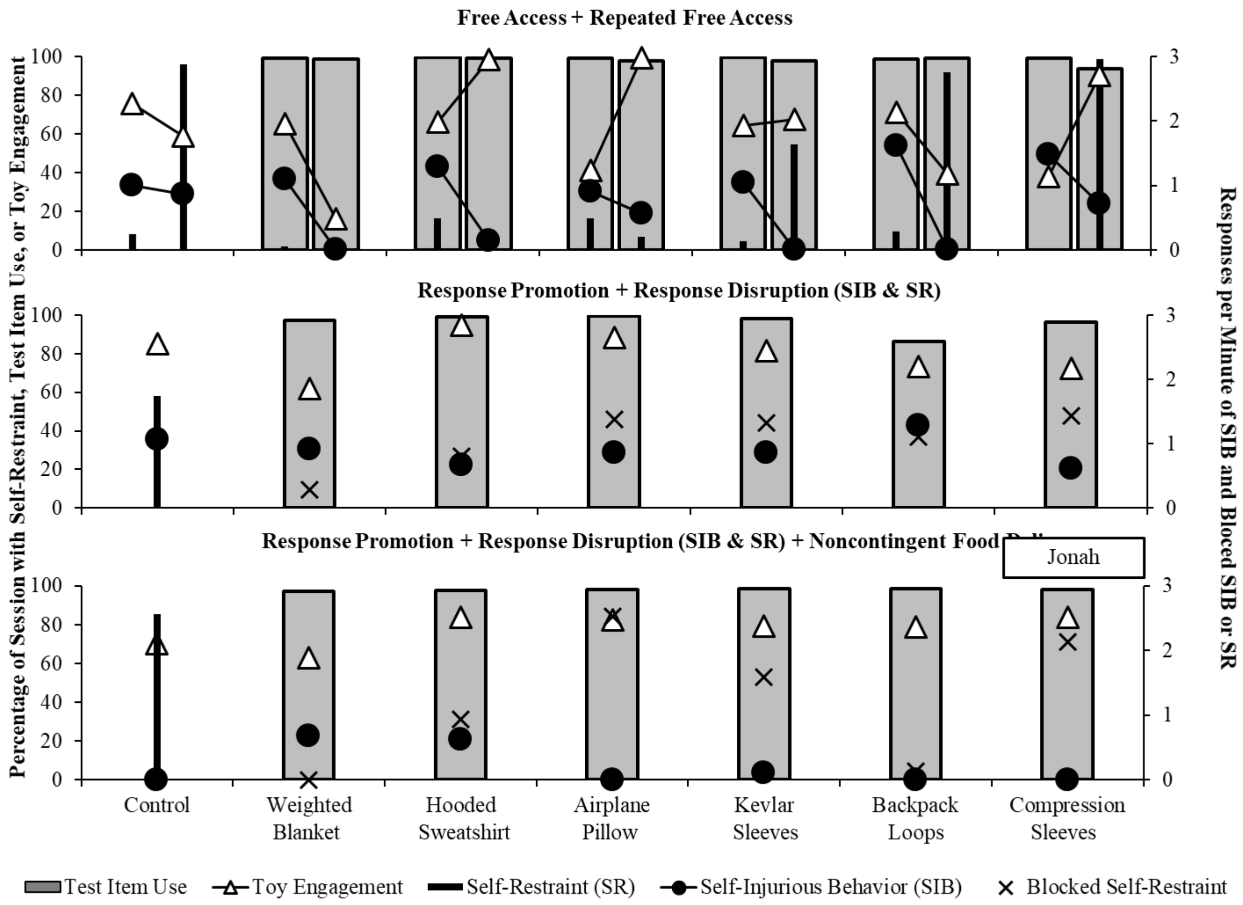

Supporting Information D

Evan's Outcomes Separated by Self-Restraint and Self-Injurious Behavior

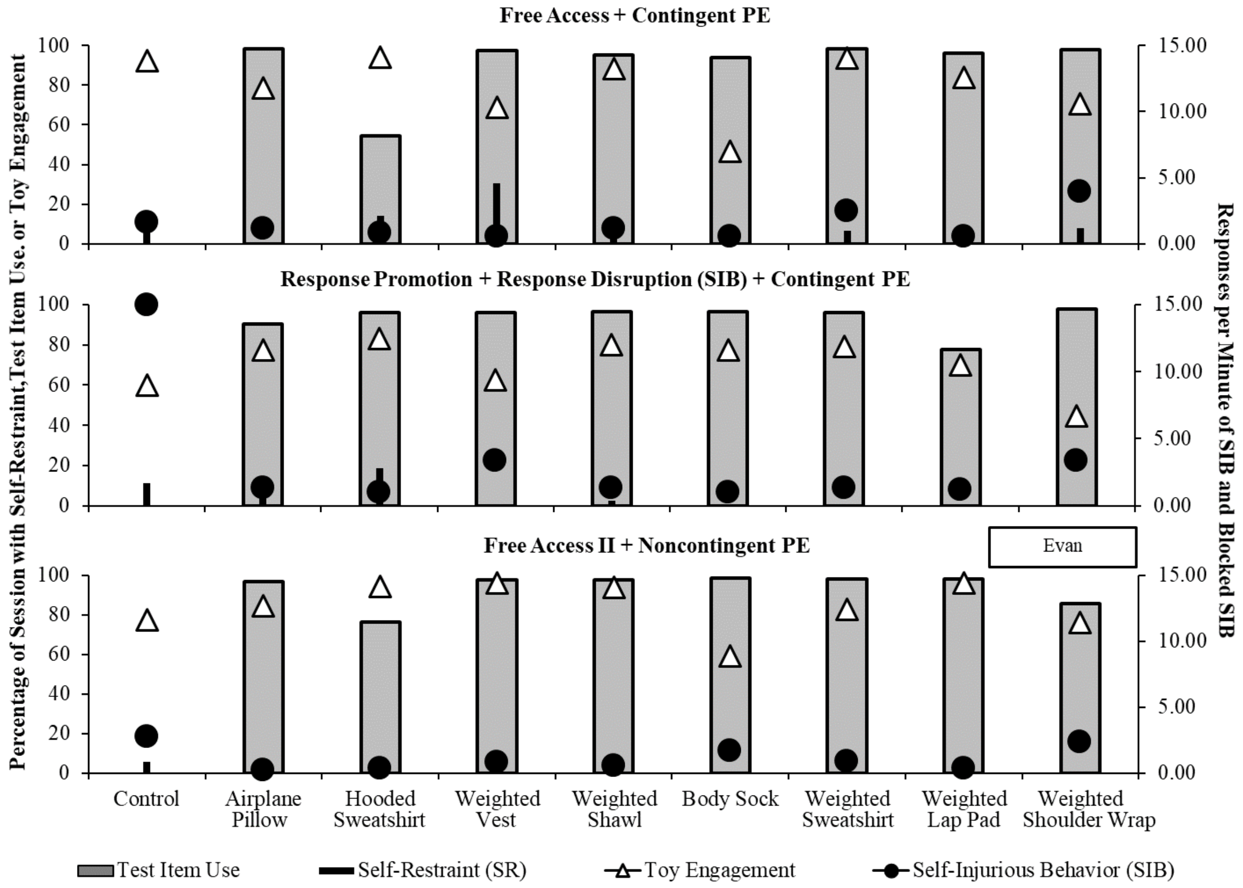

Supporting Information E

Zuri's Outcomes Separated by Self-Restraint and Self-Injurious Behavior

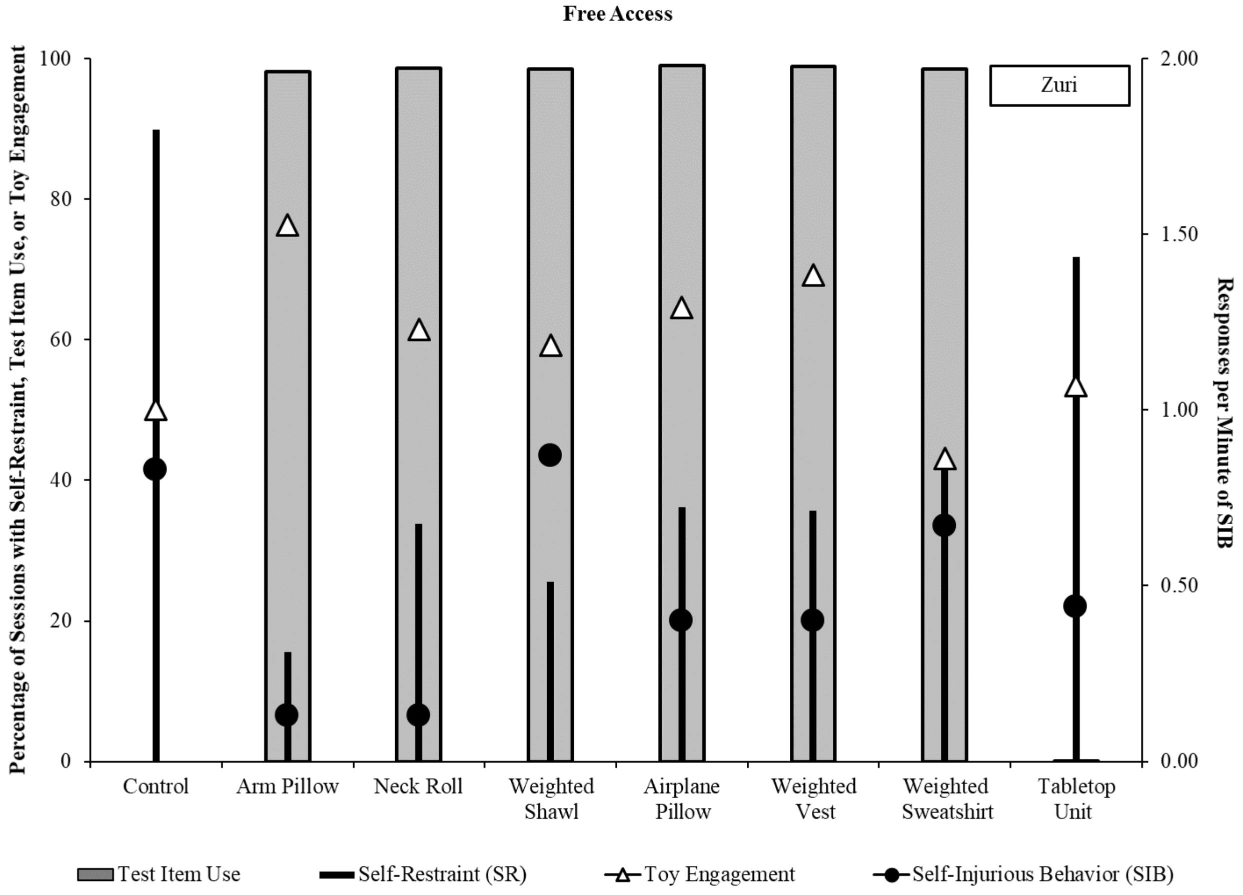

Supplement: Supplementary file 1 — Data S1: Supporting Information [file JABA-59-0-s001.pdf]
