# Supplementary material for: Application of the augmented competing stimulus assessment to identify and establish competing self‐restraint items
Source: J Appl Behav Anal. 2025 Nov 3;59(1):e70040. doi: 10.1002/jaba.70040 (PMC12689246; doi:10.1002/jaba.70040)
Supplement: Supplementary file 2 — Data S2: Supporting Information [file JABA-59-0-s002.pdf]

Supporting Information F: Safety Checklist for Documentation of Client/Participant Protections

The following checklist can be used to document the protections taken to maintain the safety and reduce the risk of injury to the client or research participant throughout the course of an assessment or intervention. The checklist should be completed at the start of an assessment, as well as updated as necessary following the inclusion of any additional protections throughout the assessment.

Participant Name or ID: Applicable to all participants unless specific participants are noted

		Specific Protection	Yes	Description/Participants
FA Methodology	Modifications to the functional analysis methodology that minimize the occurrence of self-injurious behavior (SIB)	Few and short sessions (Brief FA)	☒	Zuri—abbreviated (5 min) sessions
		IISCA or PFA	☐	
		Latency FA	☐	
		Precursor FA	☐	
		Trial-based FA	☐	
		Other functional analysis modification	☐	
FA and SCIA Procedures	Procedures specific to conducting an assessment or treatment that could increase overall safety	No-interaction condition conducted	☒	FA and SCIA control trials: No interaction used instead of alone for all participants
		Screened for automatic reinforcement	☐	
		Session pausing or breaks	☒	FA and SCIA: Session holidays, forgoing sessions due to medical reasons resulting in the inability to fully participate. Clearance to reinitiate sessions provided by medical staff
		Open-contingency class	☐	
		Special topography selection	☐	
		Specific termination criteria	☒	FA and SCIA: Individualized termination criteria developed for all participants; further, we terminated sessions early if there was concern for safety to the participant or those in session
		Other session/topography modification	☒	SCIA: Abbreviated trial durations; trial duration was individualized to maximize efficiency and decrease risk to participants with higher rates of SIB
Environmental Safety	Modifications made to the physical environment, to monitoring of sessions, or as a response to SIB to increase safety	Covert observation	☐	
		Medical exam/clearance	☒	FA and SCIA: Participants visited on-site nursing if sessions were terminated. Medical staff indicated whether sessions could continue or needed to be paused
		Padding (to test area or therapist)	☒	Session areas were padded or softened using the following items:<br>• FA: Nash—padded room; Jonah—padded room; Evan—padded room; Zuri—portable mat, padded room; Daniel—padded room<br>• SCIA: Nash—floor mat, portable mats, padded room; Jonah—soft sheet on surfaces, floor mat, portable mat, padded room; Evan—padded room, floor mat, portable mat; Zuri—floor mat, portable mat, padded couch; Daniel—floor mat, portable mat
		Response blocking	☒	Specific behaviors that placed the participant at risk of significant injury (e.g., eye-poking, head-banging) were always blocked and included:<br>• FA: Nash—head banding (HB), neck SIB; Jonah—HB; Evan—Head-SIB and HB; Zuri—HB; Daniel—HB<br>• SCIA: Nash—Neck-SIB, HB; Jonah—head-SIB, HB, knee-to-head SIB, face rubbing; Evan—Head-SIB and HB; Zuri—head-SIB, HB, self-biting, knee-to-head SIB; Daniel—HB
		Sanitary procedures	☐	
		Sessions monitored by medical staff	☐	

		Other environmental modification	☐	
Ethical Protections	Procedures related to protections of human subjects in biomedical and behavioral research	Caregiver consent	☒	FA: Caregivers provided ongoing vocal consent for patients to complete an FA prior to participation in the study<br>SCIA: Caregivers provided written informed consent for participants to participate in the study involving the SCIA
		Institutional review board approval	☒	SCIA: Study was approved by an IRB
		Participant assent	☐	
		Other ethical protection	☒	SCIA: The study received regular oversight by an external Data Safety Monitoring Board comprised of three behavior analysts, one of whom was also a developmental pediatrician; they were not affiliated with the study. Approximately every 6 months, the Data Safety Monitoring Board received a report detailing all study sessions conducted, safeguards in place, and any adverse events that occurred during the study (no adverse events occurred)
Staffing Protocols	Procedures specific to therapists in session that could be associated with increased safety	Multiple therapists in session	☒	SCIA: A second therapist was in sessions for Jonah and Zuri to increase the likelihood that blocking of SIB could be accomplished consistently
		Parent or teacher as therapist	☐	
		Parent/teacher in room/observing	☐	
		Pre-session pairing	☐	
		Reviewed cost-benefit analysis of FA	☐	
		Specific training in safety	☒	FA and SCIA: All staff working with the participants were trained to competency in behavior management skills to safely and appropriately block and redirect behavior as needed
		Staff in close proximity	☒	FA and SCIA: Staff remained within arm's reach of the participants at all times
		Other staffing protocol	☐	
Protective Equip.	Items that decrease risk of injury caused by SIB but do not restrict movement	Protective equipment	☒	Protective equipment worn noncontingently across all sessions included the following:<br>• FA: Nash—knee pads; Evan—padded helmet; Zuri—Kevlar sleeves, foot guards<br>• SCIA: Nash—knee pads; Evan—contingent weighted hat (initial free access; applied following 10 instances of head-SIB) and noncontingent weighted hat (free access 2; applied noncontingently at the start of each trial); Zuri—biker gloves, Kevlar sleeves
		Protective clothing	☒	Various forms of protective clothing were worn noncontingently across all sessions and included the following:<br>• FA: Nash—long pants and long sleeves shirt<br>• SCIA: Zuri—compression leggings
		Other protective items	☐	
Self-Restraint	Self-restraint items used voluntarily and associated with reduced levels of SIB	Permitted occurrence of self-restraint	☒	FA: Permitted self-restraint during functional analysis and some phases of SCIA (see Procedures)
		Provided access to self-restraint items	☐	
		Other self-restraint items	☒	SCIA: Designed to evaluate test items to facilitate an adaptive self-control response to replace self-restraint or compete with SIB. These items were available during test conditions

**Mechanical Restraint**	Items applied to the individual that restrict movement to prevent the occurrence of SIB	Arm splints	☐	
		Arm immobilizers	☐	
		Posey mitts	☐	
		Other mechanical restraint	☒	Restraint items worn noncontingently across all sessions included the following:<br>• FA: Zuri—wrist guard
